# A call for congressional action: revisiting the U.S. coordinated framework for the regulation of biotechnology

**DOI:** 10.3389/fbioe.2025.1702481

**Published:** 2025-11-20

**Authors:** Leah Buchman, Emma Kovak

**Affiliations:** 1 Novonesis A/S, Washington, DC, United States; 2 Georgetown University, Washington, DC, United States; 3 The Breakthrough Institute, Berkeley, CA, United States

**Keywords:** biotechnology, CRISPR, innovation, coordinated framework, agriculture, national security, GMO, gene editing

## Abstract

Since the 1986 release of the Coordinated Framework for the Regulation of Biotechnology almost 40 years ago, there have been two whole of government updates that made only minor changes, while new regulations and guidance from individual agencies have made more substantial alterations. Despite scientific advances and the emergence of products that fall outside the purview of legacy statutes, repeated calls for substantive changes have gone largely unanswered. We expand upon the NSCEB’s most important recommendations for improvements to the Coordinated Framework. We recommend that Congress create an NBCO and direct it to create a centralized application submission portal; conduct horizon scanning for future products of biotechnology; streamline regulations for familiar products and exempt low-risk products; and improve organizational structure, staff training, and interagency exchange.

## Introduction

### Lead up to the Coordinated Framework

The emergence of recombinant DNA (rDNA) technology in the early 1970s marked the beginning of a new era in molecular biology, enabling scientists to isolate, cut, and recombine genetic material across species boundaries. As researchers began to explore the applications of these new techniques, ethical, environmental, and safety concerns were raised about their potential implications. During the 1973 Gordon Research Conference on Nucleic Acids, leading scientists expressed apprehension over the unknown consequences of rDNA experimentation. In the aftermath, a group of prominent researchers sent a letter to the National Academy of Sciences, Engineering and Medicine (NASEM), calling for a temporary moratorium on certain types of rDNA research and urging the development of safety protocols (Berg et al., 1974).

The call for safety protocols led to the 1975 Asilomar Conference on rDNA, a seminal event where over 100 scientists, lawyers, and journalists gathered to develop consensus guidelines for the safe conduct of rDNA research (Berg et al., 1975). The resulting “Asilomar Guidelines” emphasized containment, risk assessment, and self-regulation, establishing a precedent for proactive governance of emerging biotechnologies. These guidelines were quickly institutionalized when the National Institutes of Health (NIH) issued the first version of the *NIH Guidelines for Research Involving Recombinant DNA Molecules* in 1976, formalizing oversight for federally funded genetic engineering research (NIH, 1976).

While early regulation focused largely on containment within laboratory settings, rapid advances in biotechnology throughout the late 1970s and early 1980s, along with the increasing involvement of private industry, raised new questions about environmental release and commercialization. The 1980 Supreme Court decision in *Diamond v. Chakrabarty* allowed genetically engineered organisms to be patented, a watershed moment in biotech regulation (Diamond v. Chakrabarty, 1980). It spurred a wave of commercial investment and accelerated the push toward field testing and market deployment of genetically modified organisms (GMOs), including agricultural crops and microbial products.

By the early 1980s, developers were requesting approval to conduct field trials of genetically modified organisms, but no unified federal framework existed to assess their potential environmental or public health impacts. A series of reports and initiatives attempted to fill this gap (NRC, 1984; NRC, 1987). At the same time, legislative attention intensified. Senators Albert Gore Jr. and Bill Owens proposed a more structured public oversight regime, emphasizing transparency, public trust, and inter-agency coordination (Gore and Owens, 1984).

### The Coordinated Framework

The developments above culminated in the 1986 release of the *Coordinated Framework for the Regulation of Biotechnology* (hereinafter referred to as the ‘CF’), published by the White House Office of Science and Technology Policy (OSTP, 1986). The CF was not a new regulatory statute but rather a policy interpretation of how existing federal laws, such as the Federal Insecticide, Fungicide, and Rodenticide Act (FIFRA), the Federal Food, Drug, and Cosmetic Act (FDCA), the Toxic Substances Control Act (TSCA) and the Plant Protection Act (PPA) would apply to biotechnology products. The CF primarily allocated responsibility among the Environmental Protection Agency (EPA), the Food and Drug Administration (FDA), and the U.S. Department of Agriculture (USDA). The CF emphasized a product-based approach, asserting that oversight should be based on the characteristics and risks of the final product, not the method used to produce it. The CF was designed to promote innovation by leveraging existing statutory authorities rather than creating new biotechnology-specific legislation.

In 1992, OSTP issued an update of the CF that reiterated the product-based focus and affirmed that existing laws were sufficient to ensure safety. The update also refined the criteria for risk assessment and stressed that regulatory review should be commensurate with risk, while reaffirming the need for inter-agency coordination (OSTP, 1992). A second major update was released in 2017 and called for improved clarity, transparency, and public engagement but did not fundamentally alter the legal or institutional architecture of the framework (OSTP, 2016; OSTP, 2017).

**FIGURE 1:**
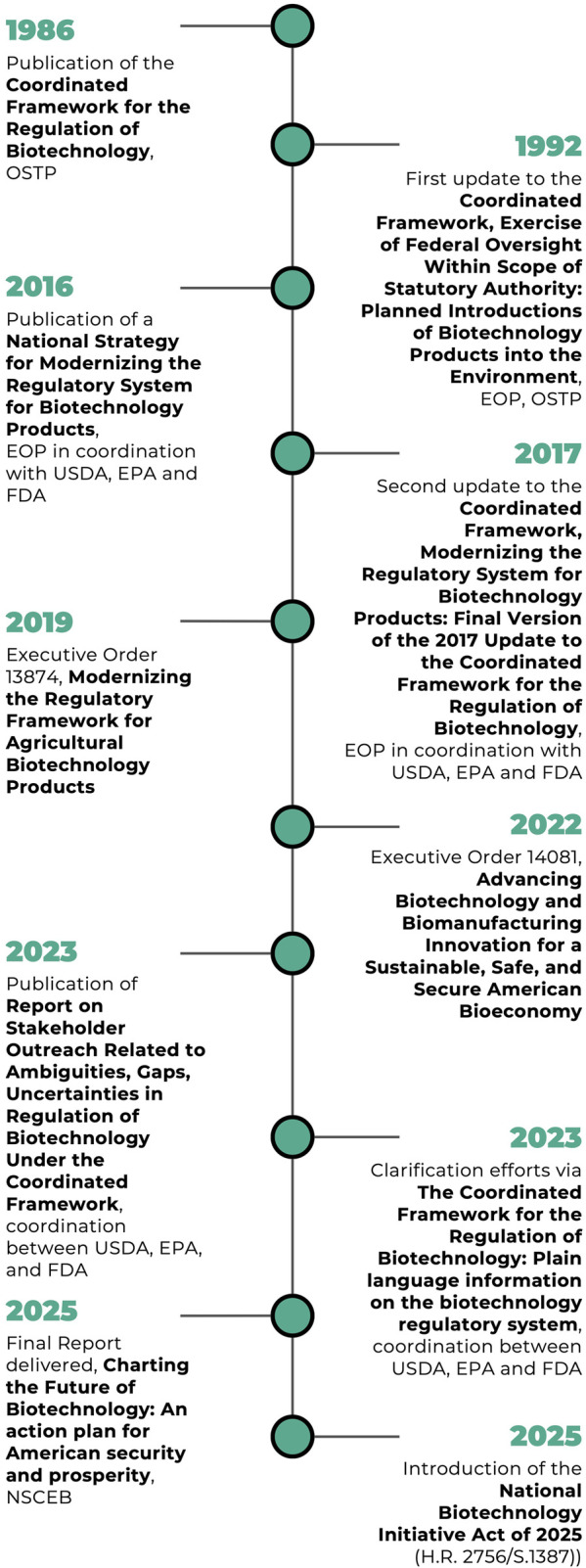
Timeline of changes to the Coordinated Framework (CF) and enabling actions.

Researchers have debated that while the 1992 and 2017 updates introduced new language, refinements in scope, and procedural guidelines, they did not meaningfully revise the underlying regulatory logic or redistribute agency responsibilities (Kuzma et al., 2008; Ahmad et al., 2024). The USDA, EPA and FDA have reasonably effectively adapted their statues to cover a wide range of products. However, the core reliance on pre-existing statutes has limited the framework’s adaptability to novel biotechnologies that fall outside the clear purview of legacy laws, such as engineered gene drives. Despite repeated calls for greater interagency coordination and updated regulatory processes, no significant legislative overhaul has occurred, and the 2017 CF remains the foundational policy instrument for biotechnology regulation in the U.S. (Figure 1).

### Executive-led biotechnology recommendations and actions

Recently in the U.S., biotechnology regulation has been shaped significantly by presidential administrations, reflecting evolving priorities around innovation, safety and global competitiveness. In 2019, President Donald Trump signed Executive Order (EO) 13874, which emphasized reducing regulatory burdens and promoting American leadership in agricultural biotechnology (USDA, 2019). The order directed agencies to adopt risk-proportionate, science-based regulation and to eliminate duplicative or overly burdensome steps in the approval process. This EO supported USDA’s efforts to implement the *SECURE* rule, finalized in 2020, which revised the agency’s oversight of genetically engineered organisms; the *SECURE* rule was vacated in December 2024 following a district court’s decision on a lawsuit challenging the rule (National Family Farm Coalition v. Vilsack, 2024).

President Joe Biden’s 2022 EO 14081 marked an expansion in the scope of biotechnology administration interest (Federal Register, 2022). Reflecting both economic and security concerns, it positioned biotechnology not only as a field of scientific inquiry but as a strategic sector critical to supply chain resilience, national defense, and climate mitigation, calling for a comprehensive bioeconomy strategy, updated regulatory paradigms for emerging biotechnologies (e.g., synthetic biology, engineered microbes), and cross-agency coordination to foster safe, ethical innovation.

### The National Security Commission on Emerging Biotechnology (NSCEB) and the case for institutional modernization

Congress created the National Security Commission on Emerging Biotechnology (NSCEB), a bi-partisan and bi-cameral commission charged with reviewing advancements in biotechnology and assessing how they shape activities by the Department of Defense. The NSCEB frames biotechnology as a national asset integral to economic security, defense preparedness, and global competitiveness. Aimed at identifying concrete steps, NSCEB developed recommendations through stakeholder engagement, and in its final report, released in April 2025, called for a more centralized and proactive governance model to ensure that the U.S. maintains leadership in a rapidly evolving technological landscape (NSCEB, 2025).

Historically, biotechnology and agricultural innovation have garnered bipartisan support in the U.S., reflecting a shared recognition of their vital roles in scientific readiness and global competitiveness (Dunn and Panetta, 2017; NSCEB, 2025). Advances in genetic engineering have been prioritized for their capacity to enhance crop resilience, boost productivity, and contribute to sustainable agriculture amid escalating climate challenges (NASEM, 2016). Moreover, the increasing recognition of agriculture and food systems as strategic national security concerns, fueled by geopolitical instability and supply chain vulnerabilities, underscores the imperative for coherent, science-based governance that balances innovation with environmental stewardship (The White House, 2022; NSCEB, 2025; USDA, 2025). Robust and proportional regulatory oversight can help the U.S. sustain its leadership in agricultural biotechnology while addressing both economic and security priorities (NSCEB, 2025).

## Policy options and implications

The NSCEB report recommends approaches to improving biotechnology governance at the federal level, and Congress should follow these recommendations. This section expands upon the most important recommendations from the NSCEB.

### Establish a National Biotechnology Coordination Office (NBCO)

Since the 1986 release of the CF, subsequent updates have yet to substantially alter coordination mechanisms at a high level, though agencies have made progress in coordinating regulation of specific types of products. Considering repeated calls for more coordination between agencies, the time is ripe to learn from past experiences and chart a new path forward.

One early coordination mechanism, the Biotechnology Science Coordinating Committee (BSCC), was established in an OSTP policy (50 Fed. Reg. 47,174 (1985)) ahead of the 1986 CF and ceased to function by 1989 (Shapiro, 1990). It was composed of senior staff from the five main agencies involved in biotechnology regulation and funding of biotechnology research: USDA, EPA, FDA, NIH, and NSF. The purposes of the BSCC as laid out in the 1985 OSTP policy were (50 Fed. Reg. 47,174 (1985):

1. Serve as a coordinating forum for addressing scientific problems, sharing information, and developing consensus
2. Promote consistency in the development of federal agencies’ review procedures and assessments
3. Facilitate continuing cooperation among federal agencies on emerging scientific issues
4. Identify gaps in scientific knowledge

During its tenure, the BSCC struggled with conflict, some of which centered around the issue of whether some organisms should be exempt from regulation. When EPA proposed its new TSCA regulations, other agencies in the BSCC disagreed with EPA’s approach and asked the Office of Management and Budget (OMB) to block the new regulations (Jaffe, 1987; Shapiro, 1990). Since the BSCC did not have statutory authority to perform its coordinating functions, it was not able to resolve this conflict between the agencies (Shapiro, 1990). To preclude similar issues from surfacing with an NBCO, one important component is clear statutory authority for the NBCO’s existence and for it to perform the specific functions outlined in the sections below. The involvement of the NBCO represents an opportunity to have fit-for-purpose regulations and guidance documents when statutory changes may not be necessary. The agencies’ roles and responsibilities and involvement with the NBCO should also be codified to support implementation by agency leaders, including the role of General Councils from each agency to help decide which agency will regulate a novel product (Carter, 2023).

In April 2025, in parallel with the report publication, Congress introduced the National Biotechnology Initiative Act, H.R. 2756 (2025); S.1387 (2025), which seeks to codify several key recommendations from the NSCEB report. The bill would establish an NBCO within the Executive Office of the President (EOP), led by a presidentially appointed director tasked with coordinating biotechnology policy across federal agencies. The legislation also directs the development of a National Biotechnology Strategy, to be updated every 4 years, with a focus on economic competitiveness, regulatory efficiency, national security, and workforce development. By embedding these priorities in statute, the Act would provide durable, institutional support for a cohesive, whole-of-government biotechnology policy, reinforcing the strategic vision articulated in NSCEB’s final report, and addressing past issues with the BSCC.

The call for a centralized authority in Recommendation 1.1a of the NSCEB report—a proposed National Biotechnology Coordination Office (NBCO)—would provide enduring executive leadership and strategic alignment across defense, commerce, health, and agriculture, addressing long-standing criticisms of fragmented oversight and regulatory inconsistency. After decades of calls for improved coordination, the NSCEB report’s proposal for the NBCO stands out as substantive and actionable.

In the following sections we describe actions the NBCO should take to facilitate streamlining of regulations.

### Centralized regulatory application submission portal

One recommendation that is not discussed in the NSCEB report is a centralized regulatory application submission portal to streamline review of biotechnology products and lessen the burden on developers. The NBCO should run such a portal, which should allow developers to submit information on a product, receive in return a list of information required for their submission package that includes requirements from all relevant agencies, and to upload the completed submission to a centralized portal. As much as possible, agencies should develop shared data standards that allow developers to submit data in one format that can be used by multiple agencies rather than submitting multiple versions of similar data required by different agencies.

A single point of entry into the regulatory system would simplify risk assessment and management by directing products to the appropriate agencies and increasing transparency for developers and the public (NASEM, 2017; Chemla et al., 2025). Beyond serving as a submission platform, the portal would act as a collaborative workspace where agency reviewers can communicate directly with each other about a product. Shared dashboards and document repositories would facilitate interagency dialogue, allowing reviewers to exchange questions, clarify data needs, and coordinate risk assessments without always needing to route communications through the developer.

Additionally, status tracking, automated notifications, and centralized documentation would improve accountability and provide developers with clear guidance throughout the regulatory process.

This coordinated approach is particularly important given overlapping jurisdictional responsibilities for biotechnology products. For instance, genetically engineered microbes used in agriculture may fall under multiple agencies: USDA-APHIS regulates under the Plant Protection Act (PPA) if the organism contains plant pest elements or could impact plant health; EPA’s Office of Pesticide Programs (OPP-BPPD) oversees microbial biopesticides under the Federal Insecticide, Fungicide, and Rodenticide Act (FIFRA); EPA’s Office of Pollution Prevention and Toxics (OPPT) regulates “new” microbial strains under the Toxic Substances Control Act (TSCA) if environmental exposure is possible; and FDA regulates food or feed applications associated with fermentation co-products under the Federal Food, Drug, and Cosmetic Act (FFDCA).

### Streamline regulation: exempt low-risk products from unnecessary regulation

There is a critical need to modernize the regulatory landscape for products of biotechnology. Policymakers and industry stakeholders are reaching a growing consensus that the current regulatory environment often stifles innovation without yielding added meaningful safety benefits (Karsten and West, 2017; BIO, 2023). Regulations should be streamlined to apply oversight “only where the risk posed by the introduction is unreasonable,” or the reduction in risk is greater than the cost (OSTP, 1992). The NBCO could ensure that USDA, EPA, and FDA regulations follow this principle.

Streamlining regulatory processes for well-understood products could also help lower barriers to entry for startups and academic ventures while maintaining rigorous oversight where unreasonable or genuinely novel risks are present. For example, FDA has conducted New Plant Variety (NPV) consultations for 81 insect resistant crops and 99 herbicide tolerant crops from 1995 to 2024 without substantially streamlining review for products similar to those the agency has previously reviewed. As a NASEM consensus report on genetically engineered crops concludes, premarket regulations should apply to “plants that express traits that are new to established, cultivated crop species and that pose a potential for environmental harm, regardless of the process used” (NASEM, 2016). Agencies should ensure their regulations exempt low-risk products, streamline regulations for organisms without new traits, and build in processes that enable agency flexibility as science and the agency’s understanding and experience evolve.

### Horizon scanning for future products of biotechnology

To remain competitive on a global scale and foster innovation, it is essential that regulatory structures anticipate emerging technologies and adapt proactively rather than reactively. The NBCO should be able to horizon scan, identify new technologies, assess how they fit into the existing CF, and highlight any concerns to Congress (Dooley, 2018). Historically, regulatory agencies have responded more reactively to new technologies. For example, CRISPR-Cas9 was first used as a gene editing tool in 2012, but USDA did not finalize the SECURE rule to incorporate gene edited plants until 2020, EPA finalized regulations for gene edited plant incorporated protectants (PIPs) in 2023, and FDA issued guidance for gene edited New Plant Varieties (NPVs) in 2024.

Regulators must prepare for the next generation of novel products, many of which may not fit neatly into existing regulatory paradigms. Recommendation 2.1b of the NSCEB report suggests that Congress instruct federal agencies to proactively develop review pathways, anticipating both the complexity of products and their societal implications. Developing anticipatory governance mechanisms, including precompetitive testing frameworks for new products, adaptive risk assessment tools, and providing agencies flexibility to assess and exclude products from existing regulations as needed will be critical to avoiding regulatory lag and maintaining public trust.

The NBCO and biotechnology regulatory agencies should partner with the NASEM, which frequently conducts horizon-scanning activities to identify emerging scientific and technological trends, such as the widely cited consensus report *Preparing for Future Products of Biotechnology* (NASEM, 2017).

### People, training, and interagency exchange

There are several additional recommendations in the NSCEB report that would support interagency coordination and streamlining of biotech regulation including formal leadership roles, bioliteracy, and interagency biotechnology training.

Effective biotechnology governance depends not only on robust processes and systems but also on the people who implement them. A key step toward improving coordination and policy coherence is addressed in Recommendation 1.2a of the NSCEB report, which suggests designation of agency biotech policy officials. These formal leadership roles would help overcome personality and organizational challenges that can impede interagency collaboration by providing clear points of accountability and communication. Even if regulations remain unchanged, the degree of effort, engagement, and coordination can vary significantly depending on the expertise and commitment of individual employees. For this reason, codifying the CF is essential to ensure consistent implementation and legally define the scope of collaboration regardless of staffing changes. Intra-agency coordination, including career staff, political appointees, and agency divisions should be prioritized as well.

Bioliteracy—the understanding of biotechnology science and policy—is critical across the federal workforce, especially as personnel inevitably change over time. The NSCEB highlights related workforce imperatives in Recommendation 5.1a, suggesting that Congress direct the Office of Personnel Management (OPM) to provide interagency biotechnology training to build and maintain critical expertise. This change would enhance regulatory capacity, support regulatory streamlining of well-characterized biotechnology products, and provide regulators with opportunities to learn about emerging technologies.

It is important to acknowledge that agencies such as USDA, EPA, and FDA have demonstrated exceptional coordination over the past several years, successfully navigating complex regulatory challenges and engaging constructively with stakeholders (USDA, EPA and FDA, 2023; USDA, EPA and FDA 2025). Sustaining this momentum requires formalizing leadership structures and ensuring knowledge sharing to promote interagency exchange. This approach will strengthen U.S. capacity to regulate biotechnology effectively and preserve the nation’s competitive edge in this rapidly evolving field.

## Actionable recommendations

Actionable recommendations are listed in Table 1.

**TABLE 1:** Actionable recommendations.

Recommendation	Involved entities	Reasoning	NSCEB final report recommendations
Establish NBCO	Legislative Branch, Executive Branch	Creating an NBCO responsible for biotechnology regulation and oversight would enable seamless interaction between agencies. An integrated system would modernize biotechnology regulation, making it more efficient, predictable and transparent while maintaining rigorous safety and environmental standards	Recommendation 1.1a urges Congress to establish a National Biotechnology Coordination Office (NBCO) within the Executive Office of the President, led by a presidentially appointed director, to coordinate interagency efforts concerning biotechnology competition and regulation
Appoint senior biotech leadership in agencies	NBCO, USDA, EPA, and FDA	Designate senior officials in each agency responsible for biotechnology governance and interagency coordination. Dedicated biotechnology leaders embedded within agencies will help institutionalize best practices, sustain bioliteracy, and support coherent policy execution	Recommendation 1.2a proposes that Congress require each relevant federal agency to designate a senior official responsible for leading biotechnology policy
Centralized application submission portal	NBCO, Regulated Industry, USDA, EPA, and FDA	Develop and manage a unified portal for biotechnology product submissions. This would help to reduce duplicative requests, align review timelines, and enable the formulation of cross-agency working groups to address complex scientific or regulatory issues	N/A
Streamlining regulation	NBCO, USDA, EPA, and FDA	Remove redundant or unnecessary regulatory steps, particularly for well understood products. This is part of ensuring that regulations yield meaningful safety benefits, meaning the reduction in risk is greater than the cost	Recommendation 2.1a calls for Congress to direct federal regulatory agencies working through the EOP to create simple, predictable pathways to market and to exempt familiar products from unnecessary regulation. The NSCEB report claims that agencies lack statutory authority to reduce regulation for well-understood products and instead must evaluate each product on a case-by-case basis whether it is familiar or novel AND Recommendation 2.1b (below)
Horizon scanning	NBCO, NASEM	Engage independent scientific bodies to assist in identifying and assessing emerging biotechnologies. Productive reports involve diverse stakeholders including from academia, small and large companies, national laboratories, and foundations	Recommendation 2.1b highlights the importance of preparing for new products of biotechnology and recommends that Congress instruct federal agencies to proactively develop review pathways, anticipating both the complexity and societal implications of future products. These biotechnologies may include synthetic organisms with no clear comparator organisms, programmable biologics, or distributed biomanufacturing platforms
Build and maintain interagency workforce capacity	NBCO, USDA, EPA, and FDA	Develop ongoing biotech training and capacity building across the federal workforce. Formalizing leadership and ensuring ongoing training and knowledge sharing can promote interagency exchange and workstream efficiency	Recommendation 5.1a suggests that Congress mandate that OPM implement biotechnology-specific training programs across federal agencies to build interagency capacity

## Conclusion

The recommendations in this article from the authors and those from the NSCEB report reflect a shift away from the reactive governance model that characterized early biotech regulation and toward a more strategic, whole-of-government approach. They echo executive actions from the last nearly 40 years that emphasized the need for a sustainable, secure bioeconomy supported by interagency coordination, infrastructure investment, and regulatory modernization (USDA, 2019; Federal Register, 2022). Yet unlike EOs, codifying these recommendations through congressional action would provide the legal and institutional permanence necessary to navigate the challenges of the coming decades.

As with earlier turning points in U.S. biotechnology governance from the Asilomar Guidelines to the CF, the NSCEB’s final report should serve as the foundation for 21st century biotechnology policy strategy. By establishing enduring structures for leadership, streamlining regulatory pathways, and preparing for the complexity of emerging products, these recommendations offer a path forward for enabling responsible innovation. Their implementation will be essential not only for sustaining U.S. competitiveness in the global bioeconomy, but for safeguarding national security in an era when biology, information, and manufacturing are increasingly intertwined.
